# Leukocyte Derived Microvesicles as Disease Progression Biomarkers in Slow Progressing Amyotrophic Lateral Sclerosis Patients

**DOI:** 10.3389/fnins.2019.00344

**Published:** 2019-04-15

**Authors:** Daisy Sproviero, Sabrina La Salvia, Federico Colombo, Susanna Zucca, Orietta Pansarasa, Luca Diamanti, Alfredo Costa, Luca Lova, Marta Giannini, Stella Gagliardi, Eliana Lauranzano, Michela Matteoli, Mauro Ceroni, Andrea Malaspina, Cristina Cereda

**Affiliations:** ^1^Genomic and Post-Genomic Center, IRCCS Mondino Foundation, Pavia, Italy; ^2^Flow Cytometry and Cell Sorting Unit, Humanitas Clinical and Research Center – IRCCS, Rozzano, Italy; ^3^Department of Brain and Behavioral Sciences, University of Pavia, Pavia, Italy; ^4^Division of General Neurology, IRCCS Mondino Foundation, Pavia, Italy; ^5^Becton Dickinson Italia S.p.A., Milan, Italy; ^6^Laboratory of Pharmacology and Brain Pathology, Humanitas Clinical and Research Center – IRCCS, Rozzano, Italy; ^7^IN-CNR, Milan, Italy; ^8^Neurodegeneration Group, Centre for Neuroscience and Trauma, Blizard Institute, Queen Mary University of London, London, United Kingdom

**Keywords:** amyotrophic lateral sclerosis, biomarkers, disease progression, microvesicles, SOD1, TDP-43

## Abstract

The lack of biomarkers in Amyotrophic Lateral Sclerosis (ALS) makes it difficult to determine the stage of the disease in patients and, therefore, it delays therapeutic trials. Microvesicles (MVs) are possible biomarkers implicated in physiological and pathological functions, however, their role in ALS remains unclear. We investigated whether plasma derived microvesicles could be overrepresented in a group of 40 patients affected by ALS compared to 28 Alzheimer’s Disease (AD) patients and 36 healthy volunteers. Leukocyte derived MVs (LMVs) compared to endothelial, platelet, erythrocyte derived MVs, were mostly present in ALS patients compared to AD patients and healthy donors. Correlation analysis corrected for the presence of confounding variables (riluzole, age at onset, site of onset, gender) was tested between PRL (Progression Rate at the Last visit) and LMVs, and a statistically significant value was found (Pearson partial correlation *r* = 0.407, *p* = 0.006). We also investigated SOD1, TDP-43 intravesicular protein level in LMVs. Misfolded SOD1 was selectively transported by LMVs and its protein level was associated with the percentage of LMVs in slow progressing patients (*r* = 0.545, *p* = 0.033). Our preliminary findings suggest that LMVs are upregulated in ALS patients and they can be considered possible markers of disease progression.

## Introduction

The discovery of disease biomarkers for prognostic purposes, clinical monitoring, and evaluation of treatment response is a major research endeavor in Amyotrophic Lateral Sclerosis (ALS), a fatal neurodegenerative disease caused by selective motor neuron death (Al-Chalabi and Hardiman, 2013; Al-Chalabi et al., 2016). Microvesicles (MVs), a subclass of extracellular vesicles, are biologically relevant, considering their cargo of RNAs, proteins, and surface receptors and they have potential to be used as biomarkers in both physiological and pathological states (Cocucci and Meldolesi, 2015). MVs (size: 100–1000 nm) are vesicles shed by budding of the plasma membrane of cells (Raposo and Stoorvogel, 2013; Lötvall et al., 2014; Cocucci and Meldolesi, 2015). They are present under physiological conditions, but they can be significantly elevated under various stimuli [increased (Ca^2+^), cellular stress, cytokine exposure, etc.] and in pathological conditions, such as cancer and neurodegenerative diseases (Yáñez-Mó et al., 2015). Upon release from their cell of origin, MVs interact only with cells that they recognize for their receptors and, once recognized, they can fuse with the plasma membrane and discharge their cargo into the cytoplasm (Prada and Meldolesi, 2016). MVs have a central role in inflammatory processes (Carandini et al., 2015) and they could be plausible targets in any research into ALS, which is characterized by an activation of astrocytes and microglia (Henkel et al., 2004; Cereda et al., 2008) as an immunological reaction to motor neuron death. Immune responses can be triggered by pathological proteins, like SOD1 and TDP-43, which have relevance to neurodegeneration (Cereda et al., 2013; Amor et al., 2014). Misfolded SOD1 is able to activate microglia by binding to the CD14/TLR4 receptor (Beers et al., 2008) and expression of TDP-43 increases pro-inflammatory markers, like IL-6, and TNFa which in glial and neuronal cells from ALS patients can act as co-activators of NF-κB (Swarup et al., 2011). SOD1 and TDP-43 can be transported by extracellular vesicles (Feneberg et al., 2014; Hanspal et al., 2017; Sproviero et al., 2018), however, it is not known whether MVs protein cargo contributes to the progression of ALS pathology. We have previously demonstrated that plasma derived MVs of ALS patients were enriched with SOD1, TDP-43, and FUS compared to controls, but we didn’t investigate if these proteins were transported by MVs of a specific origin. Zachau’s group has previously demonstrated the presence of high level of LMVs (Leukocytes derived microvesicles-CD45+MVs) in the cerebrospinal fluid (CSF) of an ALS patient (Zachau et al., 2012). Here, we investigated the role of CD45 MVs sub-typing in blood for clinical stratification of ALS patients and MVs function, as potential carrier of misfolded proteins, alternative route for disease propagation.

## Materials and Methods

### Standard Protocol Approvals, Registrations, and Patient Consents

The study protocol was approved by the Ethical Committee of the IRCCS Mondino Foundation (Pavia, Italy). Subjects participating in the study signed an informed consent (Protocol n°375/04 – version 07/01/2004). The study conformed the standards of the Declaration of Helsinki. Plasma was isolated from 40 sporadic ALS patients (SALS) (mean age at sampling: 67 ± 9.91). ALS diagnosis was made according to the revised El Escorial Criteria (Brooks et al., 2000). ALS individuals harboring mutations in the *SOD1, FUS/TLS, TARDBP, C9ORF72* were excluded from this study. Patients with concomitant infections (pneumonia and infection at the site of gastrostomy) were excluded and biological signs of inflammation present (CRP-ESR) were normal. Patients’ demographic and clinical characteristics are reported in Table 1. Progression rate at the last visit (PRL) was calculated as 48 minus the ALS Functional Rating Scale–Revised score (ALSFRS) at the last visit divided by the disease duration (in months) from onset of symptoms to the last visit (48-ALSFRS/Δt) (Lu et al., 2015). Progression rate lower than 0.5, and higher than 0.5 were defined as slow (ALS-slow) and fast progressing ALS (ALS-fast), respectively. Thirty-six sex and age-matched healthy volunteers, not on any pharmacological treatment (mean age: 51.04 ± 9.9) were used as controls (CTRL). Plasma was also collected from 28 patients affected by Alzheimer’s Disease (AD; mean age at sampling: 75.8 ± 7.3). Diagnosis of AD was based on Aging-Alzheimer’s Association work group criteria (McKhann et al., 2011). AD patients were used as neurological controls to assay if the evaluated parameters are specific of the disease. All methods were performed in accordance with the relevant guidelines and regulations.

**Table 1:** Demographic and clinical features of ALS patients.

Variables	Data
Age (years)	67.0 (SD 9.9; range:47–86)
Spinal/bulbar	32/8 (80/20)
ALS-FRSr^†^	35.8 (SD 8.5; range:6–48)
PRL^†^	Range 0.01–3.12
Fast/Slow	17/23


*^†^ALSFRS, ALS Functional Rating Scale; PRL, Progression Rate at last visit.*

### Blood Sample Collection and Isolation of MVs

Blood samples were obtained from patients with ALS and AD and healthy controls by peripheral venepuncture into BD Vacutainer^TM^ blood collection tubes with Sodium Citrate (BD Biosciences, United States). Within 1 h, blood sample was centrifuged at low speed (1,000 ×*g* for 20 min, 1600 ×*g* for 15 min) to separate plasma and remove platelets. Platelet-free plasma was then transferred to a new tube and snap frozen at -80°C. Prior to the analysis, platelet-free plasma was thawed on ice and it was centrifuged at 20,000 ×*g* for 1 h with Centrifuge 5427R (Eppendorf, Italy). The pellet was washed with 0.22 μm filtered PBS and centrifuged at 20,000 ×*g* for 1 h. The pellet was then processed for MVs analysis. Western Blot analysis for MVs marker (Annexin V, Santa Cruz Biotechnology, United States) and EXOs marker (Alix, Abcam, United States) and Nanoparticle-tracking analysis (NTA) were run to confirm MVs purity (Supplementary Figure S1) as previously described (Sproviero et al., 2018).

### Flow Cytometry Analysis of MVs

Microvesicles pellet was re-suspended in 1 ml of 0.22 μm filtered Annexin V binding buffer 1× (BD Biosciences, United States). MVs were incubated with conjugated primary antibody as listed in the antibody section. Samples were analyzed immediately after labeling, using a BD FACS Canto II with BD FACS Diva software (BD Biosciences, United States). A standardized calibrated-bead strategy using polystyrene beads (Megamix-Plus, BioCytex, France) was used, as previously described (Nielsen et al., 2014), to discriminate MVs from background noise. The polystyrene/latex beads used were a mix of fluorescent beads of varied diameters, selected to cover a theoretical MVs size range (0.16–0.20 and 0.2–0.5 μm), using SSC as a size-related parameter. Polystyrene/latex beads acquisition setting allows the cytometer to study MVs within a constant size region and getting reproducible MV counts. The range 0.16–0.20 μm was used to define the threshold background and MVs range was between 0.2 and 0.5 μm as shown in Supplementary Figure S1. MVs with positive staining for Annexin V and cell specific markers CD45 (leukocyte antigen), CD31 (endothelial cell antigen), CD61 (platelet antigen), and CD235a (erythrocyte antigen) were selected. Logarithmic amplification was used for all channels and results were referred to the percentage of Annexin V^+^ MVs, co-expressing another specific cell lineage marker, as previously described in the literature (Robert et al., 2009).

### Immunoprecipitation of Leukocyte Derived Microvesicles (LMVs)

Microvesicles were separated as described above and resuspended in PBS+BSA 1%. Anti-CD45 antibody (Santa Cruz Biotechnology, United States) was coupled to Dynabeads (Invitrogen, United States) and then incubated with MVs overnight at 4°C. Proteins retained (immunoprecipitated, IP) on the beads were recovered by adding cold Radio-Immunoprecipitation Assay (RIPA) buffer containing phosphatase and protease inhibitors (Sigma-Aldrich, Italy) and Laemmli buffer 2× and boiled at 95°C for 5 min to obtain CD45^+^ MVs. Immunodepleted fraction (I-) and input (starting material of MVs) were lysed in cold RIPA buffer, mixed with Laemmli buffer 2× and denatured at 95°C for 10 min.

### Western Blot Analysis

Proteins were fractionated by size on SDS Precast 8–16% polyacrylamide gels (BioRad, Italy), transferred to a nitrocellulose membrane using a *Trans-*blot Turbo (BioRad, Italy) and blocked with blocking solution (5% non-fat dry milk in Tween-20 Tris-Buffered Saline solution, TBS-T) for 1 h. Membranes were incubated overnight with primary antibody in blocking solution at 4°C. Membranes were then incubated for 1 h at room temperature with secondary antibodies. Antibodies used are listed in the antibodies section. Bands were visualized using an enhanced chemiluminescence detection kit (ECL Advance, GE Healthcare, United Kingdom). For subsequent immunoreactions, primary and secondary antibodies were removed from the membrane with stripping solution incubated for 20 min (100 mM Glycine, 0.1% NP-40, 1% SDS pH 2.2). Densitometric analysis of the bands was performed using ImageJ software (National Institutes of Health, United States).

### Machine Learning Analysis

Orange software (Demsar et al., 2013) was used to perform an exploratory analysis of MVs distribution in CTRL and ALS groups, with a focus on the percentages of Annexin V^+^ MVs expressing specific cell lineage markers. Logistic regression was performed and receiver operator characteristic (ROC) curves were computed (Cook, 2008). The percentages of Annexin V^+^ MVs expressing the cell lineage markers (CD45^+^/Annexin V^+^, CD235a^+^/Annexin V^+^, CD31^+^/Annexin V^+^, CD61^+^/Annexin V^+^), were used as regressors for the multivariate analysis and referred to disease phenotypes as described in flow cytometry analysis. Briefly, multivariate logistic regression was performed to predict the class of each sample (healthy donors or patients) and to rank features based on mutual information criteria (Peng et al., 2002).

### Antibodies

Antibodies, used for flow cytometry were mouse monoclonal anti-human CD45 (2D1) (ab 641417APC-H7); mouse monoclonal anti-human CD31 (MEC13.3) (ab 560983 PE); mouse monoclonal anti-human CD61 (RUU-PL7F12) (ab 347408 Per-cy); mouse monoclonal anti-human CD235a (GA-R2 (HIR2) (ab 563666 Pe-cy7) derivation and the apoptotic marker Annexin V (ab 550407 APC-BD) (BD Biosciences, United States). Immunoglobulin isotype-matched control antibodies were purchased from BD Biosciences, United States. Antibodies used for western blot were rabbit polyclonal primary antibody anti-CD45 (Santa Cruz Biotechnology, United States), mouse monoclonal anti-misfolded SOD1-DSE2-3H1 (kindly given by Prof. Neil R. Cashman) and mouse monoclonal anti-TDP-43 (Proteintech, United States). For phospho-TDP-43 we used the same antibody we utilized for the detection of TDP-43, which recognizes the intact 45 kDa protein as well as all post-translationally modified (phosphorylated and glycosylated forms) and truncated forms in multiple applications. Secondary antibodies used were donkey anti-rabbit or anti-mouse secondary peroxidase-conjugated antibody (GE Healthcare, United Kingdom).

### Statistical Analysis

The statistical analysis was carried out using Graph Pad Prism 5.0 (GraphPad Inc., United States) and SPSS statistical package version 22 (IBM, United States). One-way ANOVA with Bonferroni’s multiple comparison test was used and a value of *p* < 0.05 was considered significant. The Shapiro–Wilk test was used to test variables for normality distribution and Levene test was used to test the assumption of homogeneity. In order to determine the correlation between continuous variables, a Pearson’s correlation test was applied when the variables were normally distributed and a Spearman’s correlation test when the variables were not normally distributed. Categorical variables were tested using the non-parametric Mann–Whitney *U*-test. A partial correlation analysis (Pearson, one-tailed test) was applied to test the correlation between PRL and CD45, while correcting for the presence of confounding variables such as riluzole (Calvo et al., 2017) or other significant factors (age at onset, site of onset, gender). A Log-transformation was used for some data if they were not normally distributed.

## Results

### LMVs Are Elevated in Plasma of ALS Patients

**FIGURE 1:**
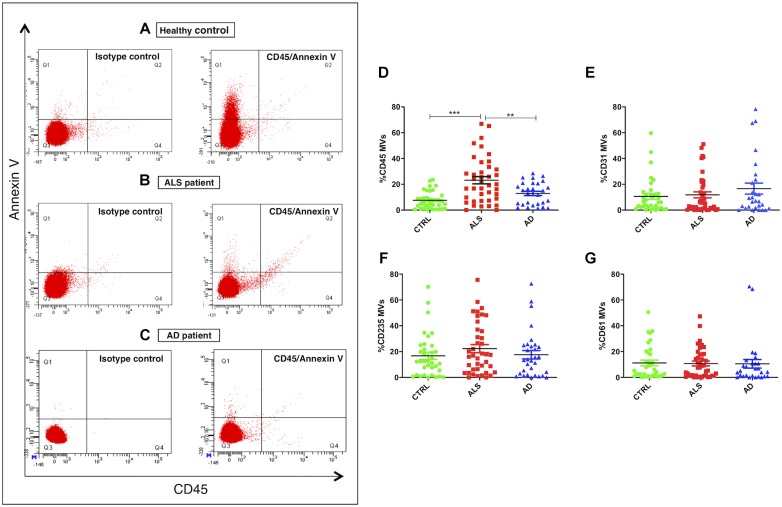
LMVs overrepresentation in plasma from ALS patients. Flow cytometry dot plots of MVs isolated from plasma of a representative healthy control, an ALS patient and AD patient labeled with Annexin V and CD45 **(A–C)**. While healthy control **(A)** and AD patient **(C)** presented few events similar to isotype control, ALS patient **(B)** had higher % CD45+/Annexin V+ MVs. Dot plots of MVs isolated from plasma of ALS patients (red), healthy controls (green), and AD patients (blue) labeled with Annexin V and CD45 **(D)**, CD31 **(E)**, CD235A **(F)**, CD61 **(G)** markers. Results were referred as the percentage of Annexin V^+^ MVs expressing the cell lineage marker. While Annexin V+ MVs derived from endothelium (%CD31 MVs) **(E)**, erythrocytes (%CD235a MVs) **(F)**, platelets (%CD61 MVs) **(G)** were not different from the healthy control subjects and AD patients (ANOVA test, *p* > 0.05), the percentage of Annexin V+ MVs derived from leukocytes (%CD45 MVs) **(D)** was significantly enhanced in the plasma of ALS patients compared to the control group (ANOVA test, ^∗∗∗^*p* < 0.001) and the AD patients (ANOVA test, ^∗∗^*p* < 0.01). *n* CTRL = 36; *n* ALS = 40; *n* AD = 28.

Correct purification of MVs was confirmed by transmission electron microscopy (TEM), NTA and the detection of classical MVs enriched markers (Supplementary Figures S1A–C) as we previously described (Sproviero et al., 2018). In Sproviero et al. (2018), we demonstrated that 90% of MVs were smaller than 500 nm by NTA. For this reason we used specific polystyrene/latex beads in the range of 0.2–0.5 μm. The percentages of Annexin V+ MVs derived from platelets (CD61), erythrocytes (CD235a), leukocytes (CD45), and endothelium (CD31) were analyzed by flow cytometry. AD patients were used as neurological controls to assay if the evaluated parameters are specific of the disease. Annexin V, that binds phosphatidylserine, is used as common marker for MVs (Lötvall et al., 2014). Flow cytometry dot plots show higher levels of LMVs in ALS patients (Figure 1A,B,D). Specifically, we observed that 75% of ALS patients had higher levels of leukocyte derived MVs (LMVs, % CD45+/Annexin V+ MVs) as percentage of all MVs subsets than the mean of the non-neurological control individuals and AD patients considered in this study as neurological controls (Figure 1C, ANOVA followed by Bonferroni’s test, *p* < 0.0001; *p* < 0.001, respectively). Conversely, percentages of Annexin V+ MVs derived from endothelium (CD31), erythrocytes (CD235a), and platelets (CD61) percentage (Figure 1E–G) of ALS patients were not different from those of healthy control subjects and AD patients. A Spearman correlation test was run among the four markers and the age (Table 2). Even if there was a significant correlation between age and CD45 MVs %, the data of the three groups didn’t meet the assumption of homogeneity of regression slopes since the Levene test was significant (*F* = 16.47, *p*-value < 0.001), so we didn’t correct for the age.

**Table 2:** Spearman correlation between CD45, CD235a, CD31, CD61% MVs, and age.

	Spearman’s rho (*p*-value) §				
Variables	CD45	CD235a	CD31	CD61	Age
CD45	1	0.384 (0.000)^∗∗∗^	0.093 (0.346)	0.117 (0.236)	0.290 (0.003)^∗∗^
CD235a	0.384 (0.000)^∗∗∗^	1	0.185 (0.060)	0.259 (0.008)^∗∗^	0.186 (0.061)
CD31	0.093 (0.346)	0.185 (0.06)	1	0.651 (0.000)^∗∗∗^	-0.023 (0.816)
CD61	0.117 (0.236)	0.259 (0.008)^∗∗^	0.651 (0.000)^∗∗∗^	1	-0.063 (0.527)


*^§,∗^Significant level, *p*-value < 0.05; ^∗∗^Significant level, *p*-value < 0.01, ^∗∗∗^Significant level, p-value < 0.001.*

**FIGURE 2:**
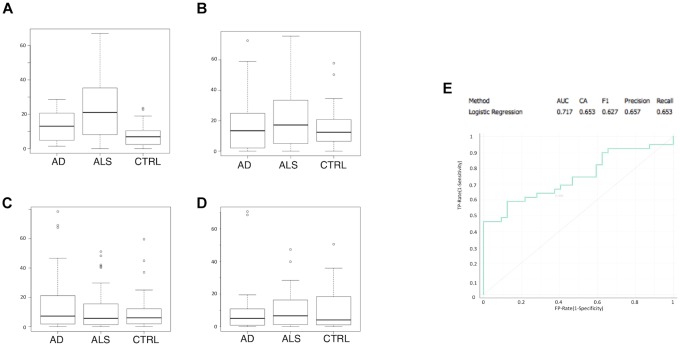
CD45+/Annexin V+ MVs is the most informative feature to discriminate ALS group from AD patients and healthy matched control groups. Boxplot of percentage of marker positive MVs are shown for ALS, AD, and control samples: **(A)** CD45+/Annexin V+; **(B)** CD235a+/Annexin V+; **(C)** CD31+/Annexin V+; **(D)** CD61+/Annexin V+; **(E)** ROC curve for logistic regression with an AUC of 0.717 and an accuracy of 0.653.

Multivariate logistic regression analysis (MLRA) was applied as a classification method for ALS and control data for the four markers (Figure 2A–D). CD45^+^/Annexin V^+^ MVs were identified as the most informative feature to discriminate ALS subgroup from AD patients and healthy controls (Figure 2A). The ROC curve (Figure 2E) showed an area under the curve of 0.717 and accuracy of 0.653 in separating patients from healthy controls. In ALS patients, no difference was found in LMV% between male and female patients (Mann–Whitney *U*-test *p* = 0.967) (Table 3). There was no statistically significant correlation between age at onset and CD45 MVs (Spearman test, *r* = 0.370, *p* = 0.072) (Table 3).

**Table 3:** Clinical and phenotypic variables effect on PRL/LMVs/SOD1.

Variables	Cases (N)	Age at onset rho (*p*-value)^§^	Bulbar_No bulbar (*p*-value)^†^	Gender_Code (*p*-value)^†^	Riluzole (*p*-value)^†^
PRL	39	0.083 (0.375)	0.878	1.000	0.043 ^∗^
CD45	39	0.370 (0.072)	0.044^∗^	0.967	0.294
SOD1	18	0.006 (0.491)	0.506	0.591	0.353


*^†^Mann–Whitney *U*-test; ^§^Spearman’s correlation test; ^∗^Significant level; *p*-value < 0.05.*

### LMVs Selective Enrichment of Misfolded SOD1

**FIGURE 3:**
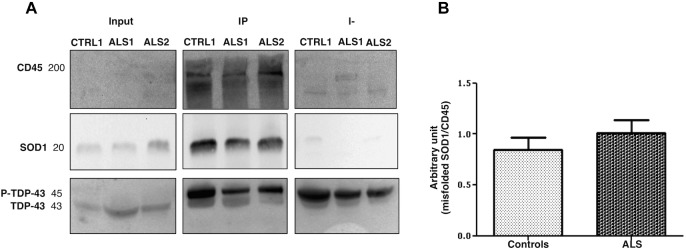
Misfolded SOD1 enrichment in LMVs (CD45+ MVs) from ALS patients and controls. CD45+ MVs of patients (ALS 1 and ALS 2) and of CTRLs (CTRL1) were enriched with misfolded SOD1. Misfolded SOD1 was only found in LMVs. The cropped blots are used in the figure and full length blots are presented in Supplementary Figure S2. The immunodepleted (I-) fraction, which include all CD45 negative MVs from other cell origin, showed no or very little level of misfolded SOD1. TDP-43 was transported in its phosphorylated form by MVs of the IP and immunodepleted (I-) **(A)**. ALS 1 and ALS 2 are referred to two slow progressing patients. Input = MVs whole lysate-20% of the IP; IP = CD45 immunoprecipitated MVs (LMVs); I- = immunodepleted. **(B)** Densitometric analysis of misfolded SOD1, normalized to CD45 band, revealed slight increase of misfolded SOD1 in all ALS patients compared to controls (0.8431 ± 0.1236, CTRL *n* = 10; 1.008 ± 0.1274, ALS *n* = 18).

We have looked at whether LMVs (the most represented MVs in blood from ALS patients), carry different levels of misfolded SOD1 and TDP-43 proteins. LMVs (CD45+MVs) were isolated from plasma of 19 ALS patients (13 = Slow; 6 = Fast) and 10 healthy controls. LMVs of all patients analyzed were enriched with misfolded SOD1 and densitometric analysis of this protein, normalized against CD45, revealed a slight increase of this protein in ALS patients compared to controls (0.8431 ± 0.1236 for CTRL and 1.008 ± 0.1274 for ALS) (Figure 3B). The immunodepleted fraction (I-), which includes all CD45 negative MVs derived from other cells, did not show any or very little level of misfolded SOD1 (Figure 3A), found mainly in LMVs.

Leukocyte derived MVs of only 12 out of 19 patients carried a TDP-43 band at 45 kDa, which resembles the phosphorylated form of TDP-43 (p-TDP-43) (Steinacker et al., 2008). TDP-43 was found in IP fraction (CD45^+^MVs) and in the immunodepleted (I-) fraction (CD45^-^MVs) (Figure 3A). These results indicate that misfolded SOD1, but not TDP-43, may be compartmentalized in LMVs.

### LMVs Correlation With Rate of Disease Progression and Misfolded SOD1 Enrichment

**FIGURE 4:**
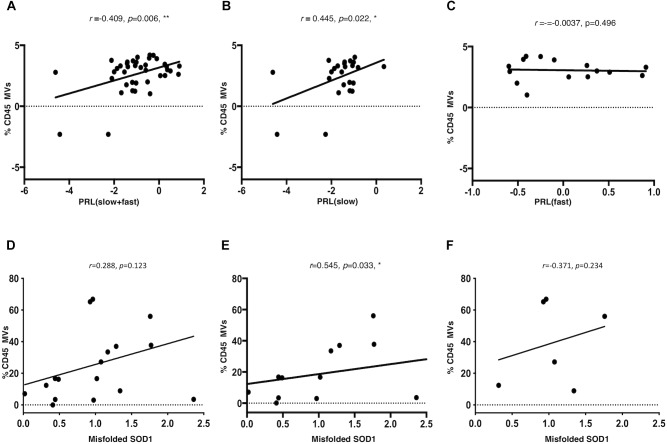
LMVs percentage in ALS patients is correlated to progression rate at last visit and is correlated to misfolded SOD1 protein level in slow progressing ALS patients. The log of PRL and of LMVs was plotted and the line was drawn from the least squares regression. A significant correlation was found between LMVs and PRL (Pearson partial correlation *r* = 0.409, *p* = 0.006) **(A)**. The PRL of slow progressing patients was positively correlated to LMVs levels (% CD45 MVs) (Pearson partial correlation *r* = 0.432, *p* = 0.022) **(B)**. On the other hand, there was no correlation between LMVs levels and disease progression in fast progressing patients (Pearson partial correlation, *r* = –0.0037, *p* = 0.496) **(C)**. CD45+ MVs percentage (%) was directly correlated with misfolded SOD1. Densitometric analysis of misfolded SOD1 was correlated to the percentage of CD45 Annexin V (%CD45 MVs) by Spearman rank analysis. LMVs level was not associated to the densitometric level of misfolded SOD1 in ALS patients (Spearman test, *r* = 0.288, *p* = 0.123) **(D)**. We instead found a strong correlation between misfolded SOD1 protein level and LMVs in slow progressing patients (*r* = 0.545, *p* = 0.033) (*n* = 12) **(E)**. No statistically significant difference was found in fast progressing patients (*r* = –0.371, *p* = 0.234) (*n* = 6) **(F)**.

A Pearson partial correlation between LMVs and PRL was evaluated, since the variables were normally distributed. The presence of a patient with a very fast progression was observed and excluded from the analysis (ALS 20, PRL = 3.12, >3sd from the mean of the PRL distribution). The cohort of patients included a number of six cases that were treated with riluzole medication, eight cases with bulbar onset and 32 with spinal onset. The effect of riluzole and site of onset on PRL were considered and corrected. Other variables were tested, but resulted not statistically significant (Table 3). A significant correlation was found between LMVs and PRL (Pearson partial correlation, *r* = 0.409, *p* = 0.006) (Figure 4A). The inclusion of the outlier in the correlation analysis did not change the results (Pearson partial correlation, *r* = 0.379, *p* = 0.009).

Amyotrophic Lateral Sclerosis patients were separated according to PRL, into slow progressing (PRL < 0.5; *n* = 23) versus fast progressing (PRL > 0.5; *n* = 17). The PRL of slow progressing patients was positively correlated to LMVs levels (% CD45 MVs) (*r* = 0.445, *p* = 0.022) (Figure 4B). On the other hand, there was no correlation between LMVs levels and disease progression in fast progressing patients (*r* = -0.0037, *p* = 0.496) (Figure 4C). The correlation between the percentage of LMVs and misfolded SOD1 was tested for 19 patients. After removing an outlier for SOD1 (>3sd from distribution mean), LMVs level was not associated to the densitometric level of misfolded SOD1 in ALS patients (Spearman test, *r* = 0.288, *p* = 0.123) (Figure 4D). We instead found a strong correlation between misfolded SOD1 protein level and LMVs in slow progressing patients (*r* = 0.545, *p* = 0.033) (*n* = 12) (Figure 4E). No statistically significant difference was found in fast progressing patients (*r* = -0.371, *p* = 0.234) (*n* = 6). We also found no correlation between LMVs and p-TDP-43 (*r* = -0.371, *p* = 0.146) (Figure 4F).

## Discussion

Microvesicles are important mediators of cross-talk among cells and are emerging as new biomarkers of neurological diseases. In this study, for the first time, we have identified an over-representation of the blood CD45+ MVs (LMVs) component in ALS patients compared to healthy controls and to AD patients. Adding to previous observations of an enrichment of CD45+ MVs in CSF from ALS patients (Zachau et al., 2012), we are hinting at LMVs as readout of innate immune response activation at a systemic level in ALS patients. This observation is relevant considering the central role that MVs occupy in any endeavor to develop biomarkers of neurodegeneration.

The LMVs may originate from neutrophils, monocytes or macrophages, lymphocytes, and can also derive from T-cells activation (Zachau et al., 2012). T-regulatory lymphocytes (Tregs) are important immunomodulatory cells that regulate the balance between activation and suppression of the immune response and control microglia activation in the central nervous system. The involvement of T-cells in ALS is supported by a host of experimental data. Treg levels in peripheral blood of ALS patients are inversely correlated with disease severity (Henkel et al., 2013) and it has been shown that rapid disease progression in ALS is associated with a higher number of differentially expressed genes in monocytes (Zhao et al., 2017).

We have found that LMVs blood expression variably correlate to PRL in ALS individuals. LMVs peripheral expression is positively linked to a slower disease progression, but not with fast disease progression. This might depend on the reported Tregs (suppressor T cells) regulation, which modulate the immune system in slowly progressing patients (Henkel et al., 2013; Murdock et al., 2016).

In ALS, extracellular vesicles have been implicated in the release and uptake of misfolded proteins mainly *in vitro* cell culture (Gomes et al., 2007; Nonaka et al., 2013; Grad et al., 2014; Iguchi et al., 2016; Pinto et al., 2017). SOD1 misfolding has been reported in all ALS sub-types, including the non-SOD1-linked familial and sporadic cases (Shibata et al., 1994) and several studies propose that misfolded protein can be both secreted and taken up by the extracellular environment through exosomes (Gomes et al., 2007; Nonaka et al., 2013; Grad et al., 2014; Iguchi et al., 2016; Pinto et al., 2017).

CD45 can bind to heparan sulfate proteoglycans (Altin and Sloan, 1997), which have been shown to be involved in aggregate uptake of prion proteins (Coombe et al., 1994; Holmes et al., 2013), and therefore, this family of receptors can be involved in the propagation of misfolded SOD1 (Gomes et al., 2007; Grad et al., 2014). In this study, we show that LMVs (CD45+ MVs) selectively transport misfolded SOD1 (but not TDP-43) and that there is a strong correlation between misfolded SOD1 protein levels in LMVs mainly in slow progressing ALS patients. This finding may suggest that slow progression may be linked to the removal of potentially toxic proteins in MVs from the blood stream. Hence LMVs may have a neuroprotective role acting as physiological scavengers of misfolded SOD1 in slow progressing ALS patients as much as in healthy controls.

We have also found p-TDP-43 in LMVs from patients, but not specifically in LMVs. Pathological TDP-43 is hyper-phosphorylated and abnormally cleaved to generate aggregation-prone C-terminal fragments (CTFs) and it can propagate from cell to cell as p-TDP-43 aggregates (Lagier-Tourenne et al., 2010). However, in our study levels of LMVs were not correlated to TDP-43, in line with previous reports. TDP-43 has been previously reported in secreted exosomes from Neuro2a cells and primary neurons, but not in exosomes produced by CD45 positive astrocytes or microglia (Iguchi et al., 2016). Our data suggest that the regulation of LMVs formation may be key to the immune response to neurodegeneration, exerting a neuroprotective effect by SOD1 misfolded protein removal, impacting on speed of disease progression. These findings might open new avenues for biomarkers discovery and strategies for therapeutics, which enhance aggregated protein clearance.

## Ethics Statement

The study protocol was approved by the Ethical Committee of the IRCCS Mondino Foundation (Pavia, Italy). Subjects participating in the study signed an informed consent (Protocol n°375/04 – version 07/01/2004). The study conformed the standards of the Declaration of Helsinki.

## Author Contributions

DS executed the experiments, designed the study, acquired and analyzed the data, drafted the manuscript, and created the figures. SLS executed the experiments, and acquired and analyzed the data. FC and LL executed the flow cytometry experiments, and acquired and analyzed the data. SZ performed the machine learning analysis and statistical analysis. OP acquired and analyzed the data. LD and MC recruited the ALS patients and analyzed the data. AC recruited the AD patients and analyzed the data. LL, SG, and EL analyzed the data. MG acquired the data. MM and AM analyzed the data and drafted the manuscript. CC conceived and designed the study, analyzed the data, and drafted the manuscript.

## Conflict of Interest Statement

LL is an employee of Becton Dickinson Italia S.p.A, which provided the antibodies used in this study. The remaining authors declare that the research was conducted in the absence of any commercial or financial relationships that could be construed as a potential conflict of interest.

## Supplementary Material

FIGURE S1 Microvesicles isolation. **(A)** Representative image of MVs (2 MVs of about 150 nm) from plasma obtained by transmission electron microscopy (TEM) (Scale bar: 100 nm); **(B)** Nanoparticle distribution by NTA confirmed the purity of MVs (mode = 137.8 +/- 6.0 nm) **(C)** Western Blot of MVs and EXOs markers in MVs and EXOs samples showed the presence of Annexin V in MVs lysate and Alix in EXOs lysate.

FIGURE S2 Western blot analysis of co-immunoprecipitation of CD45^+^ MVs with misfolded SOD1 and TDP-43 of Figure 3A. Immunoblots of MVs (input), immunoprecipitated CD45+ MVs (Bound) and of CD45- MVs (Unbound) from plasma of an healthy donor (CTRL1-lane 1, 4, 7) and two ALS patients (ALS 1-lane 2, 5, 8) and ALS 2-lane 3, 6, 9) with rabbit anti CD45 antibody **(A)**, misfolded SOD1 -3H1 **(B)**, TDP-43 **(C)**. Black squares show the cropping locations.

FIGURE S3 Leukocyte derived MVs are enriched in ALS patients with a different trend from endothelial, platelet and erythrocyte derived MVs. The histogram represents the enrichment of CD45, CD31, CD61, and CD235a MVs in four representative ALS patients and CTRLs **(A)**. Flow cytometry dot plots of MVs isolated from plasma of a representative ALS patient labelled with CD45 and CD235a **(B)** and CD61 and CD31**(C)**.

## Abbreviations

ALS: amyotrophic lateral sclerosis
EVs: extracellular vesicles
LMVs: leukocyte derived microvesicles
MVs: microvesicles
