# Supplementary figures and images for: Leukocyte Derived Microvesicles as Disease Progression Biomarkers in Slow Progressing Amyotrophic Lateral Sclerosis Patients
Source: Front Neurosci. 2019 Apr 15;13:344. doi: 10.3389/fnins.2019.00344 (PMC6476347; doi:10.3389/fnins.2019.00344)

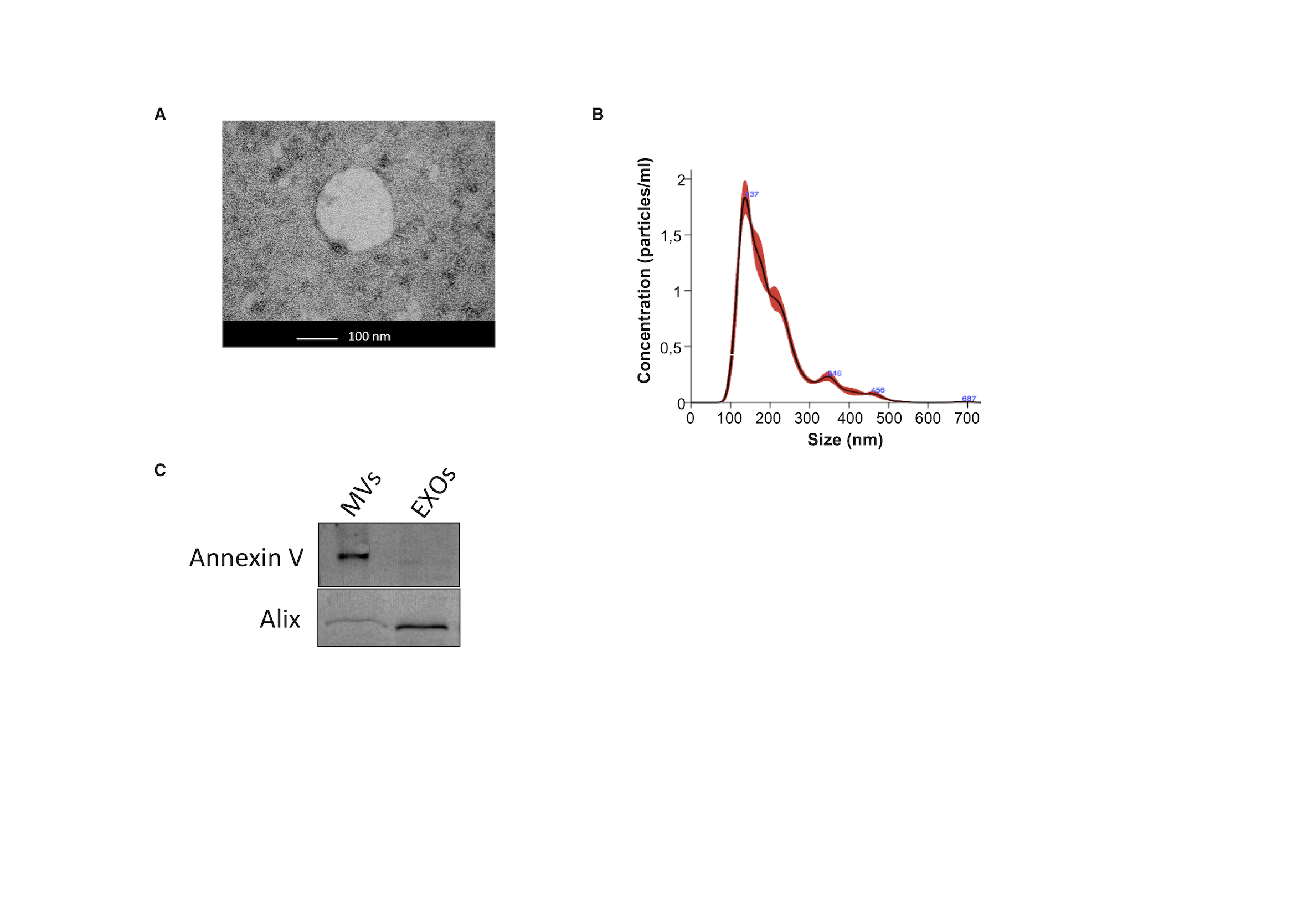

Supplement: FIGURE S1 — Microvesicles isolation. (A) Representative image of MVs (2 MVs of about 150 nm) from plasma obtained by transmission electron microscopy (TEM) (Scale bar: 100 nm); (B) Nanoparticle distribution by NTA confirmed the purity of MVs (mode = 137.8 +/- 6.0 nm) (C) Western Blot of MVs and EXOs markers in MVs and EXOs samples showed the presence of Annexin V in MVs lysate and Alix in EXOs lysate. [file Image_1.TIFF]

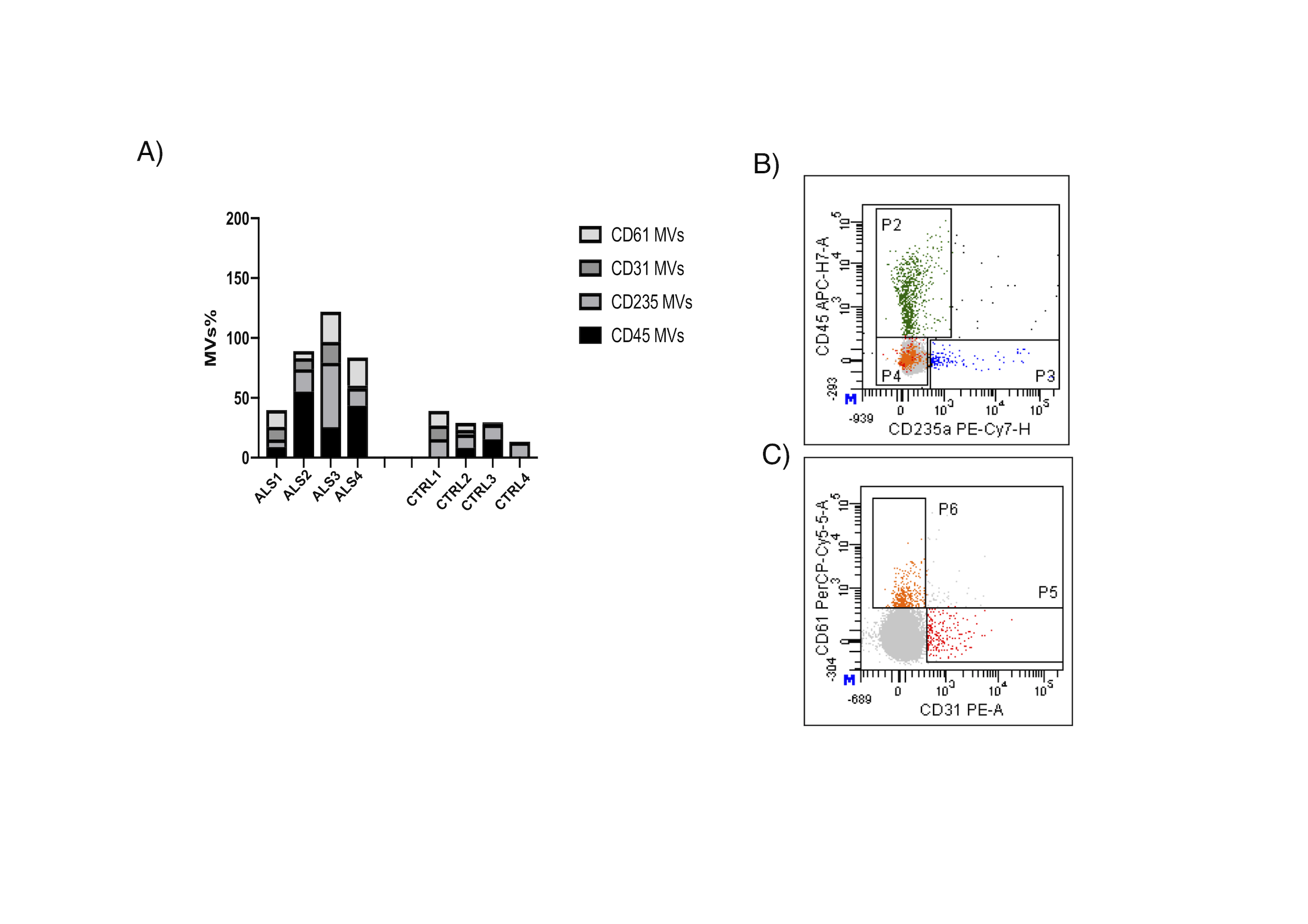

Supplement: FIGURE S3 — Leukocyte derived MVs are enriched in ALS patients with a different trend from endothelial, platelet and erythrocyte derived MVs. The histogram represents the enrichment of CD45, CD31, CD61, and CD235a MVs in four representative ALS patients and CTRLs (A). Flow cytometry dot plots of MVs isolated from plasma of a representative ALS patient labelled with CD45 and CD235a (B) and CD61 and CD31(C). [file Image_3.TIFF]
